# Complete ileal obstruction secondary to a 22-year old non-absorbable suture for an open appendectomy: Clinical case report

**DOI:** 10.1016/j.ijscr.2024.109538

**Published:** 2024-03-16

**Authors:** Yasir Alshareefy, Tala Muassess, Mahpara Mir, Hussam Altrabulsi, Ali Alshareefy

**Affiliations:** a School of Medicine, Trinity College Dublin, The University of Dublin, Ireland; b Department of Surgery, Medcare Hospital, Dubai, United Arab Emirates; c Department of Gastroenterology, Medcare Hospital, Dubai, United Arab Emirates

**Keywords:** Small bowel obstruction, Iatrogenic, Terminal ileal volvulus, Appendectomy

## Abstract

**Introduction:**

Small bowel obstruction (SBO) is a common surgical emergency. Our report describes a case of a 61-year-old male who was found to have a PROLENE suture left in situ from a previous open appendectomy 22 years ago, over which a fibrous adhesive band had formed, resulting in a terminal ileal volvulus and subsequent SBO.

**Case:**

A 61-year-old male presented with a 3-day history of severe lower abdominal cramps, nausea and constipation. A previous open appendectomy, performed 22 years ago, was the only significant detail in his medical history. A CT can with oral contrast was performed which showed dilatation of the terminal ileum and a complete absence of opacification of the cecum. Laparoscopy was then performed and a large adhesive band which formed over a suture from his previous open appendectomy was observed. On dissection of the adhesion, the bowel decompressed and returned to normal. Patient was discharged with no complications.

**Discussion:**

This is quite a unique case due to the structure of the adhesive band that was formed and the resulting terminal ileal volvulus which is an uncommon occurrence. We could not find any similar reports in our search of the literature and believe our report is novel in this regard.

**Conclusion:**

We explored a novel etiology of adhesion formation over a foreign body left in situ and this should be considered by surgeons, especially when the clinical picture is uncommon such as a terminal ileal volvulus in this case.

## Highlights

• Small bowel obstruction (SBO) is a common surgical presentation.
• Adhesions are the most common cause of small bowel obstruction.
• 61-year-old male with a previous open appendectomy presented with SBO.
• SBO caused by adhesive band formed around terminal ileum resulting in terminal ileal volvulus.
• Found to have had a PROLENE suture left in place from previous appendectomy, over which an adhesive band formed
• Important learning point that foreign bodies should be considered in the etiology of uncommon sites of SBO such as a terminal ileal volvulus in this case

## 1 Introduction

Small bowel obstruction (SBO) is a common surgical emergency, resulting in than 300,000 operations every year in the United States [1]. Adhesions are irregular scar tissue which forms between structures that should not normally be bound to each other. Some of the risk factors for the development of bowel adhesions include previous surgery, infection and trauma [2]. Adhesions are considered the most common cause of small bowel obstruction, being responsible for up to 75% of all SBO [3]. Our report describes a case of a 61-year-old male who was found to have a PROLENE suture left in situ from a previous open appendectomy 22 years ago, over which a fibrous adhesive band had formed, resulting in a terminal ileal volvulus and subsequent SBO.

## 2 Methods

This case report has been reported in accordance with the SCARE criteria [4].

## 3 The case

**Fig. 1:**
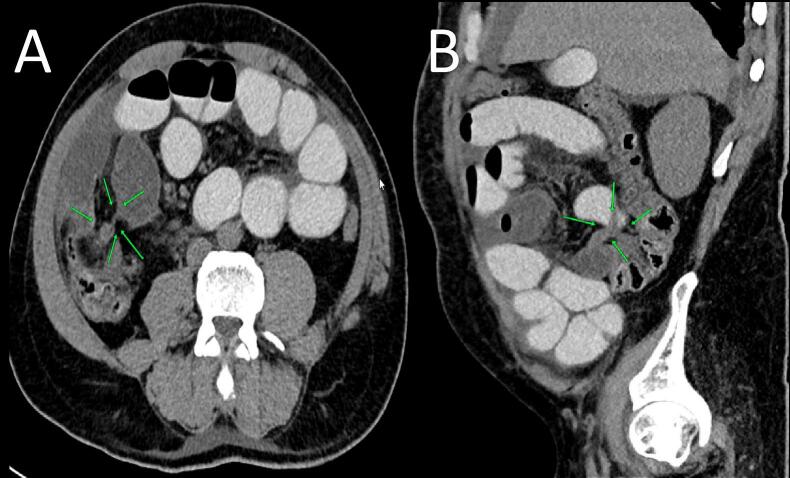
Axial (A) and Sagittal (B) CT scan showing a small segment of terminal ileal narrowing and multiple dilated small bowel loops consistent with small bowel obstruction.

A 61-year-old male presented to the emergency department with a 3-day history of severe lower abdominal cramps, nausea but not vomiting and constipation. His history revealed that he has been having ongoing intermittent post-prandial lower abdominal pains; however, it was never severe enough to warrant medical attention. A previous open appendectomy, performed 22 years ago, was the only significant detail in his medical history. In the emergency department, initial blood work including full blood count (FBC), C-reactive protein (CRP), liver function tests (LFTs) and amylase was performed, however all the results were unremarkable. A Computed Tomography (CT) with oral contrast was performed which showed significant segmental dilatation of the terminal ileum and a complete absence of opacification of the cecum (Fig. 1). These findings were discussed at a multidisciplinary team meeting including a gastroenterologist and a general surgeon and a plan for conservative management with bowel rest, intravenous hydration, analgesia and a colonoscopy to assess for luminal pathology of the cecum and terminal ileum which would influence subsequent surgical management, was agreed upon and the plan was conveyed to the patient and his family for approval.

**Fig. 2:**
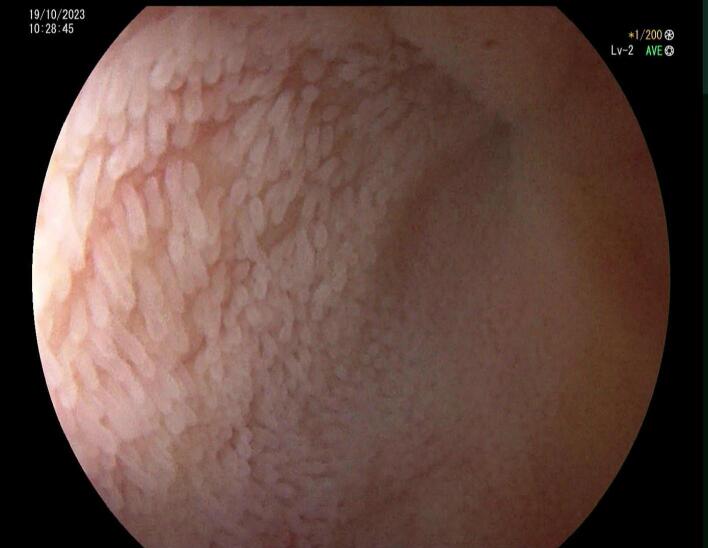
Colonoscopy showing a completely obstructed terminal ileum with a normal mucosa and no evidence of any lesions.

Following limited bowel preparation with four phosphate enemas, a colonoscopy was performed with satisfactory luminal and mucosal views to the caecum and revealed sigmoid diverticulosis, no evidence of a mass or polyp in the caecum, a normal ileocecal valve and a completely collapsed ileal lumen 10 cm proximal to ileocecal valve consistent with the CT scan findings (Fig. 2). The findings of the colonoscopy were shared with a colorectal surgeon for further planning.

**Fig. 3:**
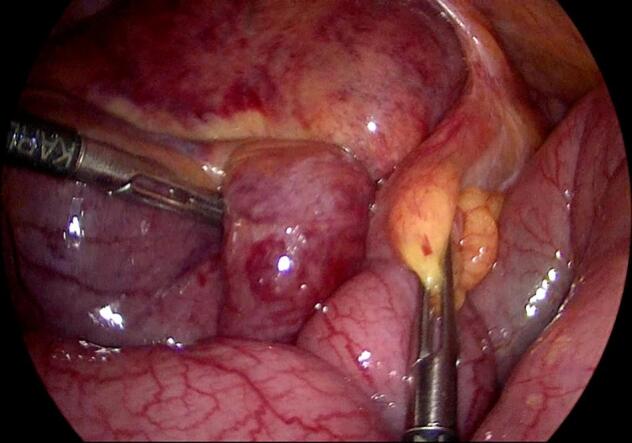
Volvulus of the terminal ileum and dusky coloured, dilated loops of small bowel.

**Fig. 4:**
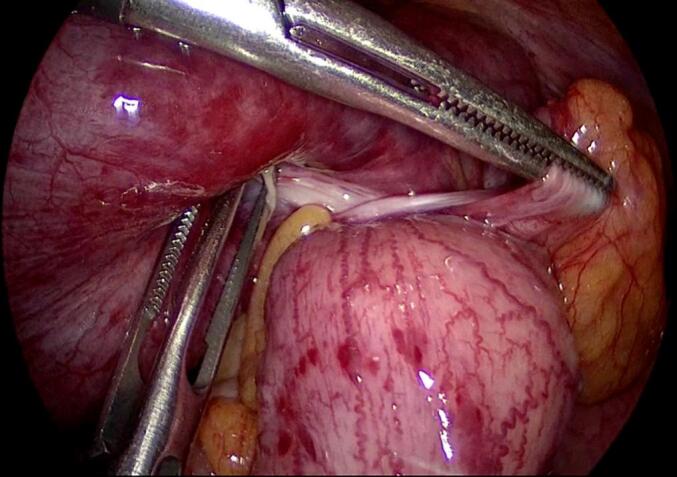
Laparoscopy showing adhesive band around terminal ileum.

**Fig. 5:**
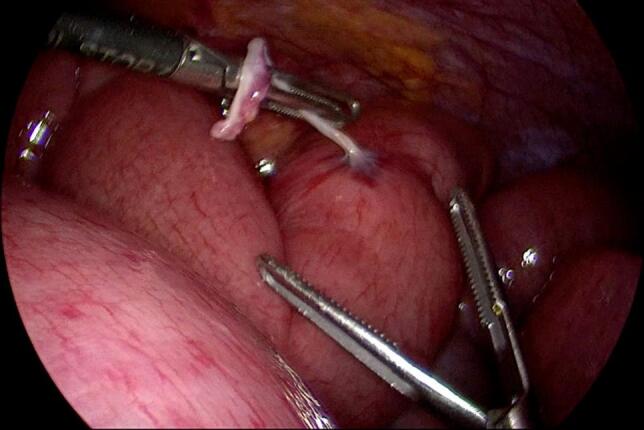
Restoration of normal colour and small bowel decompression following dissection of adhesive band containing visible suture fibres.

Entry into the abdominal cavity was gained using an optic 5 mm trocar, inserted at the left palmar point. Laparoscopy revealed a dusky coloured, twisted ileal loop, just proximal to the ileocecal junction, with dilated small bowel loops and a moderate amount of ascites in the pelvis (Fig. 3). Two more 5-mm trocars were inserted in the midline, one above the umbilicus and one in the supra-pubic area. Further exploration around the ileocecal junction revealed an adhesive band causing a volvulus of the distal ileum. The adhesive band was cut, releasing the ileal loop and resulting in decompression of the small bowel loops and immediate peristalsis. The area of ileal volvulus later regained a normal colour, indicating intact vascularity (Fig. 4). The adhesive band was removed and examined grossly; we dissected the fibrous tissue away, which revealed a suture that was enclosed in the adhesive band (Fig. 5).

In the post-operative period, the patient reported complete resolution of pain and passage of stool shortly after surgery and was tolerating water and a soft diet. He remained comfortable, did not require medication the night post-surgery, and continued to enjoy a normal diet. He was discharged then the following morning in a stable condition. The patient was subsequently followed up in clinic with no current issues.

## 4 Discussion

Appendectomy is one of the most common surgical operations performed in hospitals. A laparoscopic approach is preferred to an open approach due to the associated shorter hospital stay and surgical complications. When the procedure is completed and the surgeon wants to seal off the now excised appendix, a purse string suture technique is often deployed, using dissolvable sutures, which results in the inversion of the appendiceal stump. These dissolvable sutures tend to take 1–2 weeks to dissolve, by which time tissue healing has occurred. However, in our report, non-dissolvable PROLENE sutures were used in the initial open appendectomy 22 years ago. Over time, an adhesive band had formed over the left-over suture which resulted in a terminal ileal volvulus and subsequent SBO.

This is quite a unique case due to the structure of the adhesive band that was formed and the resulting terminal ileal volvulus which is an uncommon occurrence. We could not find any similar reports in our search of the literature and believe our report is novel in this regard.

The patient was discharged on post-op day 1 in a stable condition and was followed up in clinic with no issues.

## 5 Conclusion

The main learning points to be reinforced from this report is that old surgeries can result in current problems, as is known with adhesion formation from previous surgeries being the most common cause of SBO, however in this report we explore a novel etiology of adhesion formation over a foreign body left in situ and this should be considered by surgeons, especially when the clinical picture is uncommon such as a terminal ileal volvulus in this case.

## Ethical approval

No ethical approval required as this report describes a single case for which the patient has consented for the publication and dissemination of. No identifiable data has been reported and this report is not part of a larger research study.

## Funding

No funding for paper.

## Author contribution

Yasir Alshareefy: initial draft, figure creation, corresponding author.

Tala Muassess: initial draft, editing.

Mahpara Mir: initial draft, editing.

Hussam Altrabulsi: lead surgeon involved in case, review of paper.

Ali Alshareefy: lead clinician involved in case from diagnosis to management, writing, review of paper.

## Guarantor

Yasir Alshareefy.

## Research registration number

N/A.

## Consent

Written informed consent was obtained from the patient for publication and any accompanying images. A copy of the written consent is available for review by the Editor-in-Chief of this journal on request.

## Conflict of interest statement

No conflicts of interest.
